# Minimally invasive liver resection to obtain tumor-infiltrating lymphocytes for adoptive cell therapy in patients with metastatic melanoma

**DOI:** 10.1186/1477-7819-10-113

**Published:** 2012-06-22

**Authors:** Melissa M Alvarez-Downing, Suzanne M Inchauste, Mark E Dudley, Donald E White, John R Wunderlich, Steven A Rosenberg, Udai S Kammula

**Affiliations:** 1 Surgery Branch, Center for Cancer Research, National Cancer Institute, 10 Center Drive, Building 10 Hatfield CRC, Room 3-5930, Bethesda, MD, 20892-1201, USA

**Keywords:** adoptive cell therapy, advanced melanoma, hepatobiliary, laparoscopy, tumor infiltrating lymphocytes

## Abstract

**Background:**

Adoptive cell therapy (ACT) with tumor-infiltrating lymphocytes (TIL) in patients with metastatic melanoma has been reported to have a 56% overall response rate with 20% complete responders. To increase the availability of this promising therapy in patients with advanced melanoma, a minimally invasive approach to procure tumor for TIL generation is warranted.

**Methods:**

A feasibility study was performed to determine the safety and efficacy of laparoscopic liver resection to generate TIL for ACT. Retrospective review of a prospectively maintained database identified 22 patients with advanced melanoma and visceral metastasis (AJCC Stage M1c) who underwent laparoscopic liver resection between 1 October 2005 and 31 July 2011. The indication for resection in all patients was to receive postoperative ACT with TIL.

**Results:**

Twenty patients (91%) underwent resection utilizing a closed laparoscopic technique, one required hand-assistance and another required conversion to open resection. Median intraoperative blood loss was 100 mL with most cases performed without a Pringle maneuver. Median hospital stay was 3 days. Three (14%) patients experienced a complication from resection with no mortality. TIL were generated from 18 of 22 (82%) patients. Twelve of 15 (80%) TIL tested were found to have *in vitro* tumor reactivity. Eleven patients (50%) received the intended ACT. Two patients were rendered no evidence of disease after surgical resection, with one undergoing delayed ACT with generated TIL after relapse. Objective tumor response was seen in 5 of 11 patients (45%) who received TIL, with one patient experiencing an ongoing complete response (32+ months).

**Conclusions:**

Laparoscopic liver resection can be performed with minimal morbidity and serve as an effective means to procure tumor to generate therapeutic TIL for ACT to patients with metastatic melanoma.

## Background

In 2010, the estimated incidence of melanoma was 68,130 with approximately 8,700 deaths [1]. The annual incidence of melanoma continues to increase [1,2]. Metastatic melanoma has a poor prognosis with a median survival of 6 to 8 months and a 5-year survival of approximately 6% [3,4]. Liver metastases are diagnosed in 10 to 20% of patients with metastatic melanoma and are associated with a poor prognosis and decreased mean survival of 4.4 months [5].

The Food and Drug Administration (FDA) has approved four systemic therapies for the treatment of patients with metastatic melanoma. Systemic high-dose IL-2 has an objective response rate of approximately 15% and a durable long-term complete response rate of 4 to 5% [6]. Dacarbazine has an objective response rate of 12% with 2 to 3% complete responses that are rarely durable [7]. Most recently, two additional agents have been approved [6-11]. Ipilimumab, an antibody against the inhibitory lymphocyte receptor CTLA-4, has an approximate 7% objective response rate with an improvement in median survival of 3.6 months [8] compared to a vaccine treatment arm. Vemurafinib, a BRAF kinase inhibitor, was shown in a randomized controlled trial to have a 48% overall response rate; however, the response duration is short lived [12,13]. Thus, current approved treatment options for patients with metastatic melanoma, while promising, are associated with variable response rates and rare durable complete responders [6-11].

The National Cancer Institute, Surgery Branch, has focused on adoptive cell therapy (ACT) of autologous tumor-infiltrating lymphocytes (TIL) as a promising treatment for metastatic melanoma which has been shown in phase II trials to result in durable complete responses [14-19]. To generate a TIL infusion product, a tumor is resected to isolate TIL, which then undergo *ex vivo* expansion. The cells are then infused back into a lympho-depleted patient concomitantly with high dose IL-2. Recent data with TIL ACT have shown a clinical response rate by Response Evaluation Criteria in Solid Tumors (RECIST) in 52 of 93 (56%) patients with metastatic melanoma and a durable complete response rate of 20% [14].

TIL ACT has thus emerged as an additional effective therapy for patients with advanced metastatic melanoma. Based upon growing experience with ACT, more extensive operations to procure tumor tissue to generate TIL have been undertaken in these patients. Two recent studies evaluated the results of thoracic and hepatic metastasectomy for generation of TIL for ACT [20,21]. Both studies showed prolonged survival in patients who underwent metastasectomy and who were treated with TIL compared to those who did not receive TIL. A significant drawback from these studies is that aggressive surgical metastasectomy performed solely for the purpose of procuring TIL carries a risk of known morbidity and mortality associated with major visceral resection. Surgical complications might delay, or even preclude, eventual adoptive transfer of TIL. In this study, we describe our laparoscopic approach for hepatic tumor procurement as a minimally invasive procedure for the procurement of TIL from liver metastases.

The objective of this study was to determine the feasibility of laparoscopic liver resection as a means of obtaining hepatic TIL for the systemic treatment of patients with advanced metastatic melanoma in the Surgery Branch, National Cancer Institute, National Institutes of Health. Specifically, we sought to determine the safety of laparoscopic liver resection, examine overall patient outcome after TIL procurement and ACT, and determine if laparoscopic liver resection is an effective means in which of procuring TIL.

## Methods

### Patients

A retrospective review of a prospectively maintained database identified 22 patients who underwent attempted laparoscopic liver resection with a diagnosis of melanoma at the National Cancer Institute, Surgery Branch, from 1 October 2005 to 31 July 2011. All patients signed an institutional review board approved consent form for tissue procurement and participation in subsequent immunotherapy protocols if the patient required further systemic therapy. Inclusion criteria included pathologically confirmed melanoma, 18 years of age or older, negative serology for HIV, Hepatitis B and C, good performance status (Eastern Cooperative Oncology Group ≤2) and life expectancy greater than 3 months.

### Surgical intervention and continuing care

Complete staging was performed with history and physical examination, laboratory assessment, computed tomographic examination of the chest, abdomen and pelvis, and magnetic resonance imaging of the brain for all patients. Additional studies were performed based on individual patient assessments. Patients did not necessarily have pathologic confirmation of melanoma in the liver before the operation. Postoperative pathology confirmed melanoma in all patients. Suitability of liver resection was based on the patient’s ability to tolerate an operation, pace of disease progression and likelihood of participation in subsequent ACT protocols. The ability to render a patient with no evidence of disease was not a criterion for surgical resection. The decision to proceed with liver resection was made on a case-by-case basis after presentation at the Surgery Branch Immunotherapy conference. The decision to resect lesions using minimally invasive techniques was based on the attending hepatobiliary surgeon’s recommendations (USK).

### Laparoscopic surgical technique

**Figure 1:**
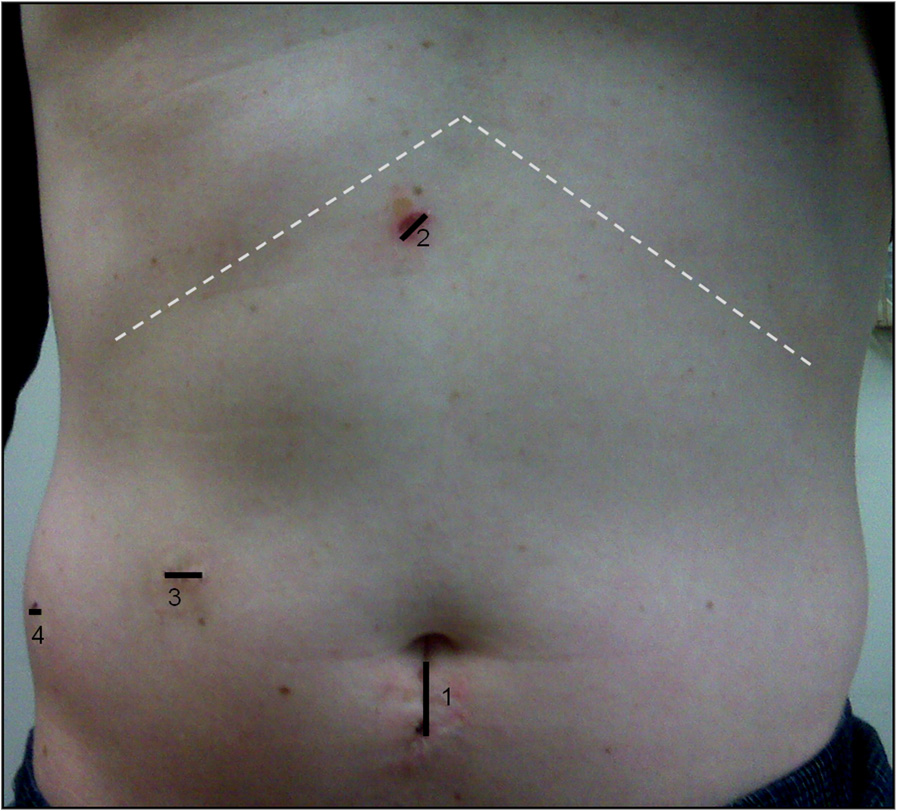
**Position of ports for laparoscopic liver resection.** The 30 degree laparoscope was inserted via a 12-mm periumbilical incision (1). A subxiphoid 5-mm port was used for liver retraction (2). Two working ports, one 5-mm and one 12-mm port, were placed along the right mid-abdomen (3, 4).

Laparoscopic resection was performed on each patient in lithotomy or supine position. Carbon dioxide pneumoperitoneum was achieved with intra-abdominal pressure maintained between 12 and 15 mmHg. A 12-mm periumbilical port was placed using the Hassan technique for a 30 degree laparoscope (Figure 1). Additional port sites were placed in triangulation to allow optimal visualization and mobilization of the liver. In most cases we used one additional 12-mm and two 5-mm ports. The initial step was to perform visual inspection of the liver followed by laparoscopic ultrasound to determine the location and extent of intraparenchymal lesions, location of inflow pedicles and draining veins, as well as to note any biliary tree involvement.

The appropriate resection line was marked on Glisson’s capsule utilizing argon electrocautery. A Pringle maneuver was not routinely performed. However, when used, an umbilical tape was passed laparoscopically around the porta hepatis and intermittent occlusion was applied as necessary. Parenchymal transection was performed with ultrasonic coagulating shears (Harmonic® Device, Ethicon Endo-Surgery, Cincinnati, OH, USA) and an endovascular stapler (Endo GIA^TM^, Covidien, Norwalk, CT, USA) when a vascular pedicle was encountered. Hemostatis of the raw liver surface was achieved with argon electrocautery and topical hemostatic agents (Floseal®, Baxter, Deerfield, IL, USA and Avitene^TM^, Bard Davol Inc., Warwick, RI, USA). The specimen was removed in an impermeable bag (Endo Catch^TM^, Ethicon Endo-Surgery, Cincinnati, OH, USA).

A low CVP (Central Venous Pressure) of 4 to 6 cmH_2_O to minimize intraoperative blood loss was maintained in each case utilizing fluid limitation, anesthetic techniques and patient positioning. The majority of surgical procedures were performed by a single surgeon (USK) with a standardized operative approach.

### Tumor-infiltrating lymphocyte production and administration

All tumors were processed in the Cell Production Facility within the Surgery Branch at the National Cancer Institute for isolation of TIL using techniques described previously [22]. Before July 2007, the activity and specificity of generated TIL were evaluated by measuring IFNγ secretion of TIL in response to a panel of tumor targets, allogeneic melanoma cell lines, fresh autologous tumor cells and peptide-pulsed T2 cells. After July 2007, cultures which successfully yielded TIL were administered without *in vitro* reactivity analysis due to concerns that these assays lacked sufficient sensitivity and specificity to accurately reflect *in vivo* efficacy [23].

TIL which met criteria for infusion underwent rapid expansion with OKT3 (anti-CD-3) antibody (Ortho Biotech, Bridgewater, NJ, USA), recombinant IL-2 (Chiron Corp., Emeryville, CA, USA), and irradiated peripheral blood mononuclear cells (PBMCs). Patients received a nonmyeloablative (NMA) chemotherapy regimen with cyclophosphamide (60 mg/kg) for 2 days and fludarabine (25 mg/m^2^) for 5 days. One patient additionally received 2 Gy total body irradiation with autologous CD34+ cells. Approximately 5 × 10^10^ TIL cells were administered to the autologous host by a 30-minute intravenous infusion after the patient completed the NMA regimen. TIL infusion was followed by 720,000 IU/kg of intravenous IL-2 every 8 hours up to 15 doses or until dose limiting toxicity was seen.

### Evaluation of response

The patients were assessed for tumor response to therapy based on World Health Organization (WHO) criteria or Response Evaluation Criteria in Solid Tumors (RECIST) every month for 6 months, then every 3 months for a year and every 6 months thereafter with radiographic imaging. Complete responders had no radiographic evidence of residual tumor after therapy. Partial responders exhibited a 30% or greater decrease in the sum of the longest diameters of their target lesions. Patients were deemed as having progressive disease if they exhibited a 20% or greater increase in the sum of the longest diameters of their target lesions or had new lesions.

### Statistical analysis

The overall survival (OS) was calculated from the date of ACT until the date of last encounter or death. The Kaplan-Meier method was used to calculate the probability of survival.

## Results

### Patient characteristics

From 1 October 2005 until 31 July 2011, 592 patients with advanced metastatic melanoma underwent surgical resection for TIL procurement. Of these, 22 patients with visceral metastasis (AJCC Stage M1c disease) were taken to surgery for attempted laparoscopic liver resection (Table 1). Eighteen (82%) patients had received prior systemic treatment before presenting to the National Institutes of Health. Of those, 11 (50%) were treated with high dose IL-2 and 10 (45%) with IFN alpha 2b. In all, 45% of patients received one previous treatment regimen, 14% received two different regimens and 23% received three or more regimens including chemotherapy, biochemotherapy, vaccines or investigational trials with targeted therapy. Only one patient had previously received ACT with gene modified peripheral blood lymphocytes. The indication for metastasectomy in all patients was procurement of TIL. Two (10%) patients were rendered no evidence of disease (NED) after surgery. One of these patients experienced disease recurrence following liver resection and received therapy with cryopreserved TIL 1 year later. Of the remaining 20 patients, 19 (95%) had extrahepatic disease. One patient had extensive liver involvement and underwent segmental resection of segment 3 to procure tumor for TIL.

**Table 1:** Clinical characteristics of 22 patients with metastatic melanoma who underwent laparoscopic liver resection for tumor-infiltrating lymphocyte harvest

**Variable**	**n**	**%**
Number of patients	22	
Age at time of resection (years)	48 (33–64)	
Sex		
Male	14	64
Female	8	36
Prior systemic treatment		
None	4	18
IL-2	11	50
IFN alpha 2b	10	45
Adoptive cell transfer	1	4.5
Chemotherapy	3	14
Biochemotherapy*	1	4.5
Vaccine	3	14
Anti-CTLA-4 antibody	2	9
Tyrosine kinase inhibitor	1	4.5
BRAF inhibitor	1	4.5
Indication for metastasectomy		
TIL	20	91
TIL/NED	2	9
Diagnosis	0	0

*Biochemotherapy = chemotherapy combined with IL-2 or IFN alpha 2b. TIL, tumor-infiltrating lymphocyte; NED, no evidence of disease.

### Operative findings and outcomes

All cases commenced with a closed laparoscopic technique. Of the 22 patients who underwent surgical resection, 20 (91%) had resection using a completely closed laparoscopic technique (Table 2). One patient required hand-assistance due to the location and inadequate visualization of the tumor in the superior part of segment 7. Another required conversion to open resection for bleeding that was uncontrolled with a Pringle maneuver. Fifty-four tumors were harvested from 22 patients. Most patients (64%) had a single liver metastasis harvested for TIL therapy, while eight (37%) patients had more than two tumors resected (up to 10). The majority of resections were either formal segmental resections (41%) or non-anatomical wedge resections (59%). There were no laparoscopic liver lobectomies performed in this series. The most common sites of procurement were from segment 3 (36%) and 6 (36%). The average size of tumor resected was 3.4 cm, with a range in size of 0.3 to 8 cm. Most procedures were performed without a Pringle (86%) and with the use of ultrasonic coagulating shears (95%) to divide the hepatic parenchyma. In addition, an endovascular stapler was used in 68% of procedures. Median estimated blood loss was 100 mL with a median operative time of 193 minutes.

**Table 2:** Operative characteristics of 22 patients with metastatic melanoma who underwent laparoscopic liver resection for tumor-infiltrating lymphocyte harvest

**Variable**	**n**	**%**
Surgical approach		
Laparoscopic, completely closed	20	90
Hand-assisted laparoscopic	1	4.5
Lap converted to open	1	4.5
No. of metastases resected	45	
1	14	64
2	3	14
≥ 3	5	23
Segment of liver resected		
Combined segment 2 and 3	2	9
Segment 3	8	36
Segment 5	2	9
Segment 6	8	36
Segment 7	1	4.5
Segment 8	1	4.5
Size (cm) of tumor resected (mean/median (range))	3.4/3 (0.3-8)	
Operative specifics (mean/median (range))		
Intraoperative blood loss (mL)	256/100 (50–1400)	
Operative time (minutes)	194/193 (90–305)	
Use of pringle	3	14
Parenchymal transection with ultrasonic device	21	95
Parenchymal transection with endovascular stapler	15	68
Duration of hospitalization (days) (mean/median (range))	4/3 (1–10)	
Postoperative complication	3	14
Bile leak	1	4.5
Rapid atrial fibrillation	1	4.5
Colitis	1	4.5

The median duration of inpatient hospitalization was 3 days, ranging from 1 to 10 days. There was no mortality associated with resection. Overall morbidity was low, with three patients experiencing a perioperative complication (Table 2). One patient developed a postoperative bile leak which was managed conservatively and resolved in 20 days. This patient required a longer hospitalization of 10 days and experienced a 1-month time delay in receiving TIL. One patient developed new-onset atrial fibrillation requiring electrocardioversion in the immediate postoperative period. There was no delay for this patient to receive TIL. Finally, one patient developed ascending colitis on postoperative day 5 of uncertain etiology. This patient was managed conservatively with antibiotic therapy and discharged home on postoperative day 7. This complication did not result in a delay to TIL therapy.

### Tumor-infiltrating lymphocyte characteristics and clinical outcomes

**Figure 2:**
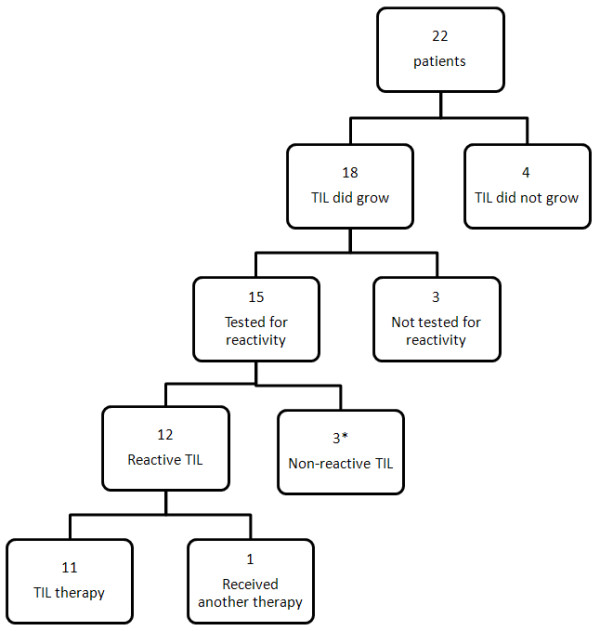
**Flow diagram of patients undergoing laparoscopic liver metastasectomy for tumor-infiltrating lymphocyte therapy.** Fifty percent of patients received the intended tumor-infiltrating lymphocyte **(**TIL) therapy. *Of the three patients without reactive TIL, one patient was rendered no evidence of disease and did not require treatment, the remaining two patients received other therapies.

Fifty-five tumors from 22 patients were analyzed for TIL testing (Table 3). Because multiple tumors can be used to generate a single therapeutic TIL treatment, all tumors harvested for a single patient were considered as a single TIL specimen. TIL cultures from 18 of the 22 (82%) patients demonstrated adequate growth (Figure 2). Of these growth-positive cultures, 15 were tested for reactivity against HLA (Human Leukocyte Antigen) matched tumor lines according to The TIL Laboratory criteria [22]. Three of the growth-positive TIL were not tested for reactivity because the respective patients were excluded from therapy due to rapid disease progression. Of the 15 TIL tested for reactivity, 12 (80%) were considered sufficiently reactive for administration as previously described [22]. In all, of the 22 patients who underwent liver resection, 11 (50%) received intended ACT therapy. Reasons to not receive TIL included rapid disease progression (3/22), TIL did not grow (4/22), TIL cultures were not reactive (2/16), resection to NED status (1/22), and enrollment onto another clinical trial (1/22).

**Table 3:** Results of tumor-infiltrating lymphocyte growth, activity and therapy

**Variable**	**n**	**%**
Number of patients with tumor resected	22	
TIL growth		
Yes	18	82
No	4	18
TIL activity		
Reactive	12	67
Non-reactive	3	17
Not tested	3	17
Treatment after metastasectomy		
TIL	11	50
No TIL	11	50
Reasons not to receive TIL therapy		
NED	1	5
TIL did not grow	4	18
TIL was not reactive	2	9
Rapid disease progression	3	16
Enrollment on another protocol	1	5
Death	0	0
Objective response to TIL		
Complete response	1	9
Partial response	4	36
No response (disease progression)	6	55
Median time (days) to receive TIL	37	
Average time (days) to receive TIL (range)	84 (35–416)	

TIL, tumor-infiltrating lymphocyte; NED, no evidence of disease.

All patients were evaluated postoperatively and were typically ready to receive systemic therapy within 1 to 2 weeks after surgical resection. One exception was in the patient who experienced a postoperative biloma. This patient was ready to receive systemic therapy 4 weeks after surgical resection.

**Figure 3:**
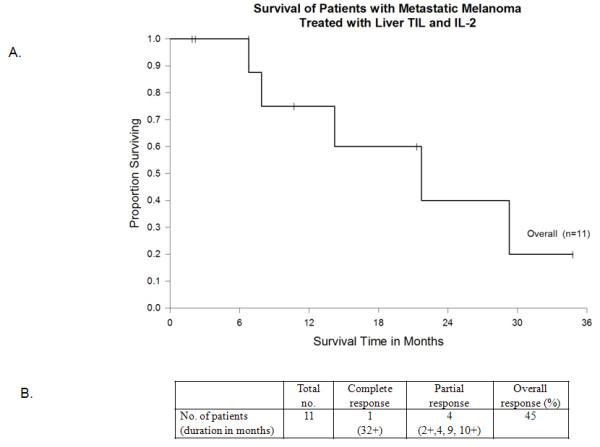
**Overall survival of patients (n = 11) undergoing laparoscopic liver resection who received postoperative tumor-infiltrating lymphocytes.** Median survival was 21.7 months.

The median follow-up time was 9.5 months for the 11 patients receiving adoptive cell therapy with TIL. Overall, there were five responders (45% response rate, 23% based on intention to treat). One patient (4.5%) had a complete response according to RECIST criteria (Figure 3). This has been an ongoing response for more than 32 months since therapy. Four (36%) patients had a partial response. Six (27%) patients did not respond to cell transfer. The median overall survival of patients receiving TIL therapy was 21.7 months.

## Discussion

Patients with metastatic melanoma have a median survival of 6 to 8 months and a 5-year survival of 6% [3,4]. FDA-approved treatments for metastatic melanoma including IL-2, chemotherapy, ipilimumab, and vemurafenib have variable response rates and rarely result in durable complete responses. With limited therapies available, efforts to develop novel regimens are critical.

The Surgery Branch at The National Cancer Institute, National Institutes of Health, has studied ACT utilizing autologous TIL as a therapy for metastatic melanoma. Recent analysis of 93 patients has shown a 56% clinical response rate with adoptive transfer of TIL [12]. Based upon these encouraging results, efforts to improve the surgical strategy for tumor procurement is an ongoing focus of the Surgery Branch. In this study, we sought to examine the role of laparoscopic liver resection to obtain tumor tissue to generate TIL for ACT in patients with advanced metastatic melanoma.

Metastases to the liver occur in 40 to 80% of patients with ocular melanoma and 10 to 20% of patients with cutaneous melanoma, making the liver a potentially important site for tumor procurement [5,10,24]. While it is optimal for a patient to undergo a low-morbidity procedure to generate TIL, in some patients liver metastases represent the only viable option to obtain sufficient tumor. In other surgical specialties, minimally invasive approaches have been found to be cost-effective and equivalent to open procedures in outcome [25-27]. In addition, these procedures are associated with decreased morbidity, decreased hospital stay and expedient postoperative recovery. Similarly, efforts examining laparoscopic versus open liver resection of benign and malignant tumors have demonstrated efficacy and safety in utilizing a laparoscopic approach [28-30]. Laparoscopic liver resection is associated with decreased hospitalization and costs [28,29,31,32], with the average postoperative hospital stay reported as 1.7 to 3.2 days [29,33-35].

Here, we retrospectively examined 22 patients who underwent planned laparoscopic liver resection to generate TIL for ACT. Successful closed laparoscopic resection was performed in 91% of patients. To be considered for resection, a patient was required to be medically stable without evidence of underlying liver disease or life threatening co-morbidities, have a good performance status, and possess favorable tumor biology. These important considerations were to ensure a patient could tolerate intraoperative hemodynamic changes associated with liver resection, as well as postoperative stress inherent to any major visceral resection. In this series, we report a morbidity of 14% with no mortality. Overall morbidity has been cited in the literature to be between 6 and 22% after laparoscopic liver resection [29,30,36-38] which is comparable with our findings. Additionally, only one case required conversion from laparoscopic to open resection secondary to bleeding, further supporting laparoscopic resection as a safe and feasible option to generate TIL.

A significant concern in laparoscopic liver resection is the ability to limit intraoperative blood loss. Parenchymal transection utilizing ultrasonic coagulating shears and an endovascular stapler for vascular pedicle division has been well established. In our experience, we found hemostasis was best accomplished utilizing the Harmonic® Device (Ethicon Endo-Surgery, Cincinnati, OH, USA) in conjunction with an endovascular stapler (Endo GIA^™^, Covidien, Norwalk, CT, USA) when a significant vascular pedicle was encountered. We did not routinely require a Pringle maneuver. With these techniques, the median estimated blood loss in our series was 100 mL, similar to that documented by others [33-35,39].

Recently, in a report of 300 laparoscopic liver resections performed over 10 years, Cannon and colleagues describe a trend towards increasingly complex resections as the authors gained experience [39]. Similarly, we initially favored anterior and peripherally located tumors for ease of resection. We avoided laparoscopic resection of tumors with a central location or close proximity to major portal or hepatic vascular structures and instead opted to resect these tumors with a traditional open approach. With experience, we have transitioned to resecting these more challenging tumors laparoscopically. Currently, we are building on our experience to perform formal laparoscopic lobectomy when this represents the sole option for generation of TIL in a given patient.

Overall, patients were typically back to daily activities and off all narcotics within 1 to 2 weeks. Only one patient experienced a delay to TIL therapy due to a complication associated with resection. This is an important finding given the intensity of the NMA preparative regimen necessary before TIL cell infusion. Thus, we conclude from this pilot experience that patients with metastases to the liver can undergo a minimally invasive procedure and receive ACT in a timely manner.

Finally, we examined the efficacy of TIL generated from laparoscopic liver resection and its translation into patient therapy and outcome. Of the patients who underwent resection, 82% had growth-positive TIL, with 12 (66%) patients demonstrating adequate reactivity to undergo expansion for ACT. This is comparable to previously published reports from the Surgery Branch [20,21]. Of note, many patients in these previous reports were subjected to major open surgical resections, such as thoracotomy, sternotomy or open hepatic resection. Of the 22 patients who underwent laparoscopic liver resection, 11 received the intended TIL therapy (50%). Overall, there was one (9%) documented complete response (duration 32+ months), four (36%) partial responders and five (45%) non-responders with an overall response rate of 45% or 23% based on preoperative intention-to-treat analysis. These response data are comparable to our previously published results of 29% and 40% response rates (8% and 25% intention-to-treat) after thoracic metastasectomy or open liver resection, respectively [14,20,40]. The median overall survival was 21.7 months. These findings emphasize that patient selection is important for successful ACT and also suggest that, as we improve upon the methods for TIL generation to treat more patients, we may further improve overall response rates.

Potential limitations of this study include the retrospective nature of data analysis and patient selection bias. Patients who are candidates for TIL therapy may represent a highly selected group with slow growing metastases and favorable tumor biology, allowing them to undergo tumor procurement and wait the necessary month for TIL generation. Further selection bias includes the selection of patients to undergo laparoscopic versus open liver resection. Despite these caveats, our current description is a valuable additional therapeutic option for patients with advanced metastatic melanoma.

## Conclusions

In conclusion, we found laparoscopic liver resection in well selected patients to be feasible with minimal morbidity and no mortality. This represents an important and effective option for patients with hepatic metastases who are candidates for ACT.

## Abbreviations

ACT: adoptive cell therapy; FDA: Food and Drug Administration; IFN: interferon; IL: interleukin; NED: no evidence of disease; NMA: nonmyeloablative; OS: overall survival; PBMC: peripheral blood mononuclear cell; RECIST: Response Evaluation Criteria in Solid Tumors; TIL: tumor-infiltrating lymphocytes; WHO: World Health Organization.

## Competing interests

The authors declare that they have no competing interests.

## Authors’ contributions

MMA is the principle investigator who prepared and wrote the manuscript. SMI provided documentation of data, literature analysis and supported the work of the principle investigator in preparing the manuscript. MED performed documentation of data and provided revisions to the manuscript. DEW performed statistical analysis and documentation of data. JRW participated in documentation of data and provided revisions to the manuscript. SAR supported the work of the principle investigator by providing revisions to the manuscript. USK conceived the study, and participated in its design and coordination and helped to draft the manuscript. All authors read and approved the final manuscript.
